# Sealing ability of lateral compaction and tapered single cone gutta-percha techniques in root canals prepared with stainless steel and rotary nickel titanium instruments

**DOI:** 10.4317/jced.50752

**Published:** 2012-07-01

**Authors:** Mustafa M. Koçak, Sis Darendeliler-Yaman

**Affiliations:** 1 PhD, DDS. Department of Endodontics, Faculty of Dentistry, Zonguldak Karaelmas University, Zonguldak, Turkey.; 2 Prof. Dr. DDS. Department of Endodontics, Faculty of Dentistry, Gazi University, Ankara, Turkey.

## Abstract

Objectives: The aim of this study was to evaluate the sealing ability of lateral compaction and tapered single cone gutta-percha techniques in root canals prepared with stainless steel and rotary nickel titanium root canal instruments by fluid filtration method. Study design: The root canals were prepared with stainless steel (SS) and nickel titanium (NiTi) instruments. The canals prepared with SS were obturated with lateral compaction technique using .02 tapered cones and the canals prepared with NiTi instruments were obturated with lateral compaction technique using .02 tapered cones or 06 tapered single cones. The amount of leakage was evaluated by fluid filtration model. The results were statistically analyzed with one-way ANOVA. Results: The group prepared with NiTi instruments and filled with lateral compaction technique showed significantly less coronal leakage than the group prepared with SS instruments and filled with lateral compaction technique (p<0.05). There was no statistically difference between apical leakages of groups (p>0.05). Conclusions: Obturation with lateral compaction of gutta-percha provides a superior coronal seal whilst canal instrumentation with engine-driven NiTi files reduces the extent of microleakage in root canals when compared with stainless steel hand instruments. Tapered single cone technique was comparable with lateral compaction technique because of easier application.

** Key words:**Apical leakage, coronal leakage, lateral compaction technique, single cone technique.

## Introduction

Instrumentation and obturation techniques are both key parts of a successful root canal treatment (1). In order to achieve an adequate three dimensional obturation, the goal of root canal obturation is to maximize the amount of solid core material and minimize the amount of sealer (2). Gutta-percha is a widely used and accepted root canal filling material. Lateral compaction of cold gutta-percha (LC) is a commonly used technique due to its simplicity and adaptability in most cases and is often used as a standard to compare new techniques (3,4). However, filling root canals by LC using a master .02 taper gutta-percha cone followed by the addition of further accessory standard gutta-percha cones is time consuming and has the potential to place undue force on the root leading to root fracture (5). Nowadays, most of the rotary Ni-Ti systems are .04 and higher tapered and the idea of a tapered gutta-percha cone became more popular. Therefore, gutta-percha cones were produced to match the taper of canals prepared with .04 or .06 rotary instruments. Since three-dimensional obliteration of the prepared canal space is the goal of obturation, the use of a master gutta-percha cone, which more closely matches the taper of the space prepared by NiTi instruments would seem to be more efficient to achieve this goal (6). Tapered cones provide three-dimensional obturation of the root canal over its whole length without the requirements for accessory cones or the additional time spent on LC (7). The use of a master cone with a larger taper increases the amount of gutta-percha within the canal, thereby reducing the amount of sealer between accessory cones, which is a desired condition to improve the three-dimensional filling of the canal (2). Additionally, obturation with a single cone requires less time. Gordon et al. (7) reported that the time of obturation for LC was significantly greater than single cone technique.

Nowadays, it is well-known that the coronal and apical leakages have important adversary effects on the long term success of endodontic treatment (8,9). Hence, the researchs about the evaluation of apical and coronal leakage characteristics of the obturation materials are important (10).

The fluid filtration method has been a widely accepted technique in the determination of apical and coronal microleakage values, which gives the possibility to evaluate the sealing ability of the very same sample in a longitudinal design (11,12).

The aim of this study was to evaluate the apical and coronal leakage of the canals prepared with stainless steel or nickel-titanium root canal instruments and filled with lateral compaction or tapered single cone techniques of cold gutta-percha.

## Material and Methods

160 freshly extracted fully developed human maxillary anterior teeth with single canal were used. The teeth were radiographed from facial and proximal orientations to confirm the presence of a single canal. A single operator performed the whole root canal preparation and obturation. After sectioning all teeth at the CEJ to provide a reproducible reference point, apical patency of each tooth was verified with a #10 K-type file and working length (WL) was established as 0.5 mm short of canal length (CL), the point at which the #10 file was first visible at the apical foramen. Considering the preparation of the root canals and obturation of the root canals 10 experimental groups were identified.

Preparation of root canal

160 root canals were instrumented. 10 roots were seperated as positive and negative controls. 30 roots were instrumented with stainless steel files to a size 40 K-file as master apical file. A crown-down technique was used and the coronal 1/3 of roots was instrumented by using Gates-Glidden drills (Dentsply, Addlestone, UK). 60 root canals were prepared with ProFile (Dentsply, Ballaigues, Switzerland) rotary instruments using a high torque motor (Endo Torque, Medidenta International, USA) set at 350 rpm. The root canals were prepared to size of 40#. The remaining 60 roots were prepared with ProTaper (Dentsply Maillefer, Ballaigues, Switzerland) rotary instruments using a high torque motor set at 300 rpm. Apical preparations was completed with ProTaper F4 file. A total of 5 mL of 2.5% NaOCl was used for irrigation of the canals. Each canal was dried with paper points.

Obturation of root canal

Lateral compaction: 30 root canals which were instrumented with stainless steel files, 30 of the 60 canals instrumented with ProFile rotary instruments and 30 of the 60 canals instrumented with ProTaper rotary instruments were obturated with cold lateral compaction technique. An ISO size 40# master gutta-percha cone was inserted into the root canal to the working. The master gutta-percha cone was then coated with AH26 (Dentsply Maillefer, Ballaigues, Switzerland) sealer and placed into the root canal. Lateral compaction with accessory gutta-percha cones was performed. Excess gutta-percha was removed with a heat source.

Single cone: 30 root canals were instrumented with ProFile and were obturated with 40# .06 tapered single gutta-percha cone (Sure-endo, Sure Dent Corp, Korea) and the remaining 30 canals were instrumented with ProTa-per and were obturated with a matched F4 gutta-percha (Dentsply Maillefer, Ballaigues, Switzerland) with AH26 sealer.

In order to evaluate the apical and coronal sealing characteristics; the fluid filtration method was utilized and during the process the equipment designed by Çobankara et al. (12) was used. The sectioned root specimens were attached to a 18-gauge stainless steel tube from the coronal side for coronal measurements and were attached to the equipment from the apical side for apical measurements 7 days after the obturation. The connections of the root and plastic tube were coated with cyanoacrylate cement circumferentially. The distilled water was filled in all the pipettes, syringes and the plastic tubes. For the measurement process, an air bubble was created in the micropipette and adjusted to an appropriate position with the syringe. Then the constant pressure was applied. The displacement of air bubble in the capillary tube was recorded as the fluid transport. The measurements were recorded at 2-min intervals for the duration of 8 min, and were averaged. The flow rate through the 18-gauge needle in an unobturated canal was determined by weighing the water mass that could flow through the 18-gauge needle in 1 min duration which was found to be 1.850 g/min at 239 cm H2O. This value served as a positive control. All values were calculated comparing the positive control group. In negative control group, the apex of each root was sealed with cyanoacrylate cement hermetically. The experiment was conducted under a standard 2 atmosphere pressure. The results was statistically evaluated by using one-way ANOVA and Tukey HSD tests.

## Results

In this study the amount of leakage observed in the positive control group was recorded in the units of ?l/cmH2O/min-1. No leakage was observed in the negative control group. Since coronal and apical leakages were considered separately, 10 groups were defined. The leakage values were shown in  Table 1.

**Table 1:**
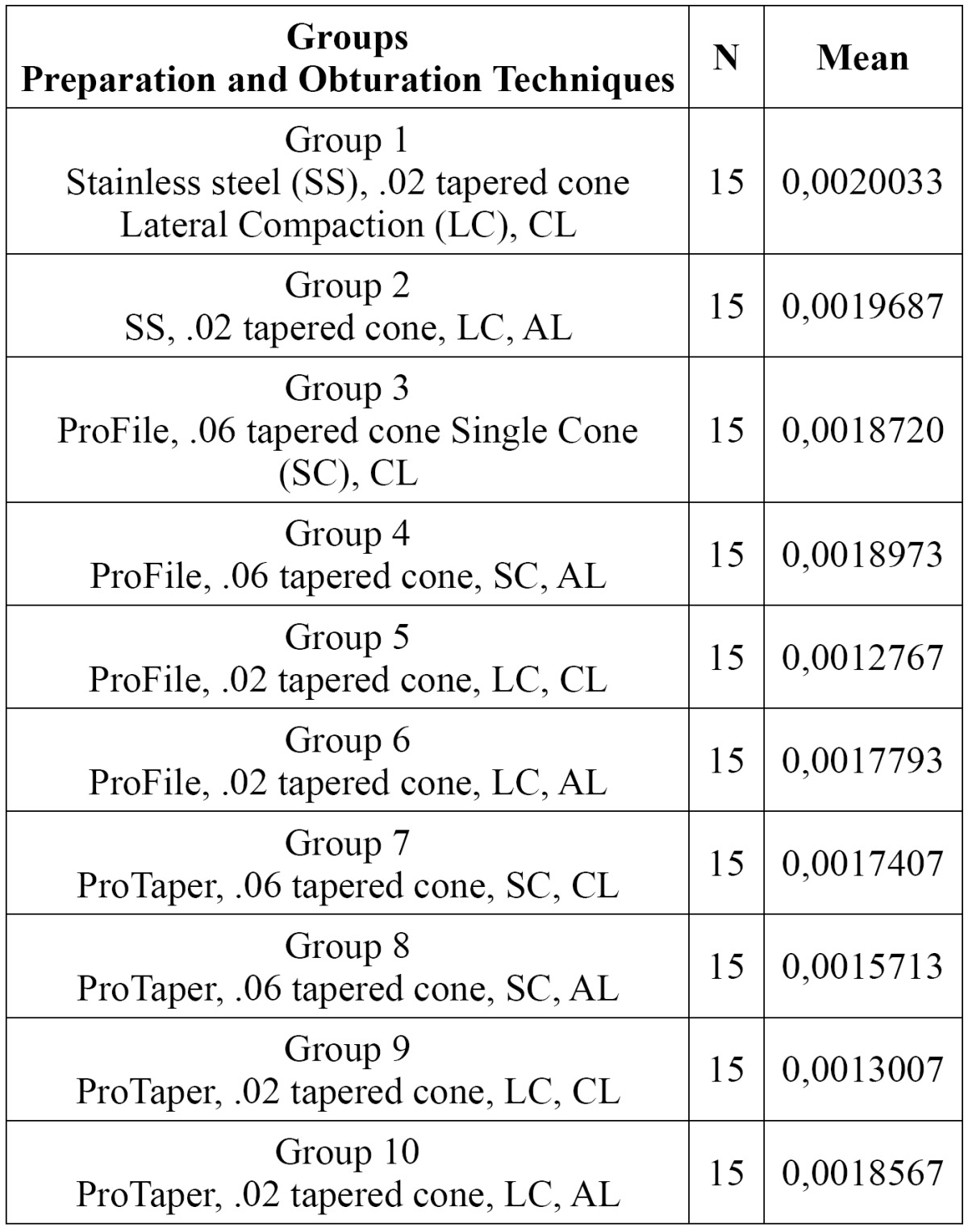
Coronal leakage (CL) and Apical Leakage (AL) Values.

Evaluation of Coronal Leakage: One way ANOVA test yielded statistically significant difference (p<0.05). According to the results of Tukey HSD test the lowest coronal leakage values was observed in specimens obturated with lateral compaction and prepared with ProFile or ProTaper rotary nickel titanium instruments. The highest amount of coronal leakage was determined in specimens prepared with stainless steel instruments and filled with lateral compaction technique. In terms of coronal leakage, the differences between the specimens prepared with stainless steel and nickel titanium instruments were found to be statistically significant (p<0.05).

Evaluation of Apical Leakage: One way ANOVA test yielded no statistically significant differences (p>0.05).

## Discussion

According to the results of this study, in terms of the coronal leakage; the groups instrumented with NiTi instruments and filled with lateral compaction technique yielded statistically significant less leakage than the group instrumented with stainless steel instruments and filled with .02 tapered gutta-percha cones. This was attributed to better canal shaping capability of NiTi instruments. Gutta-percha showed better adaptation with the canal walls and resulted in lower amount of leakage.

It is generally believed that the NiTi instruments perform better even in curved canals and produce better cleaning especially in the apical region as compared with the stainless steel instruments. Better shaping causes less irregularities and in the presence of less debris the root canal fillings produce better adaption which consequently results in less amount of leakage. Von Fraunhofer et al. (13) compared the leakage of NiTi and stainless steel instruments. The result of the study revealed that the root canals instrumented with NiTi instruments leaked less than the canals instrumented with stainless steel instruments which is similar with the result of our study.

The results of the current study also revealed that when instrumented with NiTi instruments; the groups filled with .06 tapered single cone and the groups filled with the lateral compaction technique did not show any statistically significant results. It was reported that the utilization of tapered cones compatible with the nickel titanium instruments increase the effects of the master cone. Contrary to that the widely accepted disadvantage of single cone technique is the weak adaptation capability of the single master cone at middle and coronal third of irregular shaped canals (14). The sealer will distribute and more leakage is probable. On the other hand, in oval shaped canals, the single cone technique will not increase the coronal obturation and hence a thicker sealer layer will be present. In the current study, since the oval shaped canals were used, the single cone technique did not perform superior to lateral compaction technique.

Bal et al. (15) conducted in vitro bacterial leakage experiment and compared the coronal leakage of teeth instrumented with .06 taper instruments and obturated .06 or .02 master cone and using lateral compaction technique They reported no statistically significant differences in terms of leakage characteristics of all groups. Yücel and Çiftçi (16) compared various root canal obturation techniques and reported that the bacterial penetration in canals obturated with single cone technique were more than those obturated with lateral compaction technique.

No difference was found between the apical leakage of groups. The results showed that in terms of the apical leakage there were no statistically significant differences between lateral compaction and single cone techniques. It is believed that in the case of the lateral compaction technique the spreader penetration causes satisfactory obturation and in the case of the single cone technique the cones having the same taper with the rotary nickel titanium instruments provide good apical obturation. Pérez-Heredia et al. (17) reported no statistically significant differences in the evaluation of the apical leakage of molar teeth instrumented with .06 taper nickel titanium files and obturated with cold lateral compaction technique by using .02 and .06 tapered master cone. These also suggest similar results to our findings.

According to the parameters obtained by Gordon et al. (7), single cone technique utilizing .06 tapered gutta-percha was found to be comparable to the lateral compaction technique. Furthermore they also stated that .06 tapered single cone technique produces faster root canal obturation than the lateral compaction technique. These are also similar to the results of the current study. According to the leakage values obtained in the current study, no statistically significant differences were observed between the teeth filled with the lateral compaction technique and those filled with single cone tapered gutta-percha. In contrast to those studies, Pommel and Camps (11) reported that the highest amount of leakage was probable in single cone technique.

The most important disadvantage of the single cone technique appears when the cone was not compatible with canal irregularities at the coronal and middle thirds of the canal (18). Consequently, the sealer accumulate there and in the long term the treatment becomes adversely effected. Additionally the volume of sealer required for the single cone technique is larger than the volume necessary to complete a compaction technique. In the single cone technique generally more leakage is observed than the lateral compation technique (16,18). But in the current study no statistically significant leakage differences were observed between the groups all instrumented with NiTi instruments and either filled with .06 tapered single cone or obturated through lateral compaction with .02 tapered cone.

The results of the current study proposes the tapered single cone technique as an efficient alternative to the lateral compaction technique because of shorter application time and ease in the application.
